# Leucine-Rich Repeat Kinase 2 (LRRK2)-Deficient Rats Exhibit Renal Tubule Injury and Perturbations in Metabolic and Immunological Homeostasis

**DOI:** 10.1371/journal.pone.0066164

**Published:** 2013-06-14

**Authors:** Daniel Ness, Zhao Ren, Shyra Gardai, Douglas Sharpnack, Victor J. Johnson, Richard J. Brennan, Elizabeth F. Brigham, Andrew J. Olaharski

**Affiliations:** 1 Nonclinical Safety Evaluation, Elan Pharmaceuticals Inc., South San Francisco, California, United States of America; 2 Assay Development, Elan Pharmaceuticals Inc., South San Francisco, California, United States of America; 3 Exploratory Biology, Elan Pharmaceuticals Inc., South San Francisco, California, United States of America; 4 Pharmacology, Elan Pharmaceuticals Inc., South San Francisco, California, United States of America; 5 Thomson Reuters Inc., Carlsbad, California, United States of America; 6 Vet Path Services Inc., Mason, Ohio, United States of America; 7 Burleson Research Technologies Inc. (BRT), Morrisville, North Carolina, United States of America; National Cancer Institute, United States of America

## Abstract

Genetic evidence links mutations in the LRRK2 gene with an increased risk of Parkinson’s disease, for which no neuroprotective or neurorestorative therapies currently exist. While the role of LRRK2 in normal cellular function has yet to be fully described, evidence suggests involvement with immune and kidney functions. A comparative study of LRRK2-deficient and wild type rats investigated the influence that this gene has on the phenotype of these rats. Significant weight gain in the LRRK2 null rats was observed and was accompanied by significant increases in insulin and insulin-like growth factors. Additionally, LRRK2-deficient rats displayed kidney morphological and histopathological alterations in the renal tubule epithelial cells of all animals assessed. These perturbations in renal morphology were accompanied by significant decreases of lipocalin-2, in both the urine and plasma of knockout animals. Significant alterations in the cellular composition of the spleen between LRRK2 knockout and wild type animals were identified by immunophenotyping and were associated with subtle differences in response to dual infection with rat-adapted influenza virus (RAIV) and *Streptococcus pneumoniae*. Ontological pathway analysis of LRRK2 across metabolic and kidney processes and pathological categories suggested that the thioredoxin network may play a role in perturbing these organ systems. The phenotype of the LRRK2 null rat is suggestive of a complex biology influencing metabolism, immune function and kidney homeostasis. These data need to be extended to better understand the role of the kinase domain or other biological functions of the gene to better inform the development of pharmacological inhibitors.

## Introduction

Parkinson’s disease (PD) is the second most common neurodegenerative disease, affecting 1–2% of the population over the age of 60 [1], [2]. There are currently no neuroprotective or neurorestorative therapies available for the treatment of Parkinson’s disease; therefore, delaying the progression of PD represents a critical unmet medical need. While the etiology of the disease remains largely unknown, with the majority of cases appearing to be sporadic, genetic evidence has linked several genes to an increased risk of an individual developing Parkinson’s, including the leucine-rich repeat kinase 2 (LRRK2) gene [3], [4]. The LRRK2 gene encodes a large protein that contains an ankyrin repeat region, a leucine-rich repeat (LRR) domain, a Ras of complex proteins (Roc) GTPase domain, a C-terminal of Roc (COR) domain, a kinase domain, and a WD40 domain [5]. Mutations in the Roc, COR, and kinase domains of LRRK2 are associated with late-onset PD indistinguishable from idiopathic disease, and have been implicated in familial and sporadic cases of PD [6], [7]. There is significant interest in elucidating the biological roles of LRRK2, uncovering the molecular and cellular effects of pathogenic mutations and generating treatment therapies that are based upon its modulation.

LRRK2, through its kinase and/or scaffolding function, appears to regulate a variety of cellular processes critical for homeostasis and survival; it has been shown to affect protein synthesis through its interaction with the microRNA processing molecule Argonaute [8] and modulate various aspects of the proteosomal and lysosomal function, including substrate clearance [9], aggresome formation [10], lysosomal positioning [11], and autophagy [12]–[14]. Importantly, LRRK2 over-expression also causes mitochondrial fragmentation and dysfunction, leading to increased vulnerability to oxidative stress and cell death [15]. In addition, mutant LRRK2 could induce apoptosis through activation of the MKK4-JNK [16] or the death receptor pathway [17]. These findings have advanced our knowledge regarding LRRK2 function and in how it contributes to the pathogenic mechanisms involved with Parkinson’s disease, however many basic biological questions remain unanswered. This information will be critical for developing future pharmacological intervention strategies.

Predicting safety liabilities of novel therapeutic targets is challenging prior to the availability of good tool compounds. One approach is to study genetically modified animal models. Specifically, a phenotypic analysis of knockout animals may provide insight into potential effects of pharmacological inhibition. Though there are a variety of confounding factors (e.g. developmental changes vs. transient pharmacologic manipulation; holoprotein deletion vs inhibition of one of several biological functions of the protein), there is still the opportunity to gain important insights in the targeted biology. In the case of LRRK2, data generated in several LRRK2 deficient mouse strains has indicated that kidney homeostasis is perturbed [18]–[20]. In addition, mounting evidence suggests that it has a role in the immune system [21] and may be involved with the inflammatory response associated with innate immune cells [22]–[24].

The purpose of the series of experiments presented herein was to perform a comparative analysis of LRRK2 wild type and deficient rats, characterize kidney morphology, histopathology and homeostasis as well as determine if the putative immunological changes have measurable effects *in vivo* following challenge with an infectious agent. Similar to previous reports in mice, LRRK2 deficient rats exhibited renal morphological and histopathological changes, with the novel finding that the renal biomarker lipocalin-2 (NGAL) was significantly reduced in both the urine and serum of knockout animals. Significant changes in the cellular composition of splenocytes were identified between genotypes, but these changes only translated to subtle differences in their response to a dual-infection insult in a host resistance study, where knockout and wild type animals were sequentially infected with rat adapted influenza virus (RAIV) and *Streptococcus pneumoniae*. Unique to this study was the observation that LRRK2 deficient rats experienced significant weight gain, which was accompanied by a number of metabolic and clinical pathological perturbations. Molecular pathway analysis suggested that an interaction between LRRK2 and thioredoxin-related pathways may be relevant for the kidney pathology and metabolic perturbations observed in these knockout animals.

## Materials and Methods

### LRRK2 Deficient and Long-Evans Wildtype Control Animals

Three separate cohorts of LRRK2 Knockout (KO) and wildtype (WT) rats were received from Sigma Advance Genetic Engineering (SAGE) laboratories and were maintained until 12 to 16 weeks of age. Rats were group-housed in microisolator cages and provided with certified rodent diet (Harlan Test Lab #2018) and water ad libitum. Environmental controls for the animal room were set to maintain 18 to 29°C, a relative humidity of 30 to 70%, a minimum of 10 room air changes/hour, and a 12-hour light/12-hour dark cycle. Rats were acclimated for at least three days before the start of any study. Group food and water consumption were recorded once at the end of study whereas individual body weights were measured once per week. Animals were euthanized in a CO_2_ chamber and blood was collected via cardiac puncture from each animal for clinical pathological and plasma biomarker analyses. Gross necropsy was conducted on each animal following terminal sacrifice, including an examination of the external features of the carcass, external body orifices, the abdominal, thoracic, organs and tissues (gross pathology). Tissues were weighed and subsequently fixed in 10% neutral buffered formalin. Kidneys, spleens, bone marrow and lungs were prepared for hematoxylin and eosin (H&E) staining and histopathological assessment by a board-certified (ACVP) veterinary pathologist (Vet Path Services; Mason OH). Three separate cohorts of animals were received from SAGE. Animals from cohort 1 were utilized for comparative immunophenotyping in the absence of viral or bacterial challenges. Animals from cohort 2 were utilized for water and food consumption, metabolic, clinical chemical, complete blood count, and urinary and serum biomarker and histopathological analyses. Animals from cohort 3 were used in the host-resistance study. All animal work completed at Elan complied with Elan IACUC protocols and was approved by the Institutional and Animal Care and Use Committee.

### Clinical Pathological, Metabolic and Kidney Biomarker Analyses

Rat blood was collected from 5 age-matched LRRK2 KO and WT male rats approximately 13 weeks of age. Samples were assessed for peripheral complete blood counts and plasma clinical chemical parameters within 24 hours of withdrawal (Idexx Laboratories, Fremont, CA). Plasma was isolated and analyzed in the Rat MetabolicMAP® and Rat KidneyMAP® panels (MyriadRBM; Austin, TX). Urine was collected on wet ice using metabolic cages for 5 age-matched LRRK2 KO and WT male rats approximately 13 weeks of age. Urine was filtered using a 0.2 micron sterile filter and analyzed for the presence of lipocalin 2 (NGAL), osteopontin, albumin and KIM-1 using the MesoScale Discoveries rat kidney injury panel (Catalog number K15162C-4; Gaithersburg, MD). Urine kidney biomarker data were normalized using creatinine levels.

### Flow Cytometric Immunophenotyping

Rat spleens from WT and LRRK2 KO rats were removed during necropsy. Splenocytes were individually isolated from half of the spleen of each animal. To enrich for WBC’s, RBC were lysed using RBC lysing buffer (eBiosciences; San Diego, CA) at RT for 15 min. WBC’s were washed with cold PBS twice, then blocked with PBS/50% rat serum at RT for 20 min. WBC’s were counted using the Vi cel (Beckman Coulter; Brea, CA), and plated at approximately 40 k/well of in a 96-well V-bottom plate. Surface antibodies (ms anti-rtCD3-PE, msIgG3-PE, ms anti-CD11b/c-PE, msIgG2a-PE, ms anti-rtCD4-FITC, msIgG2a-FITC, ms anti-rtCD8-PE, msIgG1-PE, ms anti-ox6-FITC, and msIgG1-FITC) were prepared at 2 µg/ml in PBS/10% rat serum (Sigma Aldrich; St. Louis, MO). Splenocyte WBC’s were spun down in the V-bottom plate, buffer discarded and cells resuspended in the respective cell marker and the isotype control solutions at 100 µl/well. After 30 min on ice, cells were spun down and washed with cold PBS twice. Cells were resuspended in 200 ul cold PBS and assessed on the LSRII flow cytometer (BD Biosciences; San Jose, CA) and the relative percentage each cell sub-population comprised within the spleen was calculated.

### Rat-adapted Influenza Virus and *Streptococcus pneumoniae* in vivo Host Resistance Study

LRRK2 KO male rats and corresponding age-matched wild type (WT) Long Evans male controls, along with Long Evans male rats used as *S. pneumoniae* infection controls, were assessed for their immunological response in a dual infection host-resistance study (Burleson Research Technologies; Morrisville, NC). Rats were acclimated for one week prior to the beginning of the experiment. All animal work completed at Burleson Research Technologies (BRT) complied with BRT IACUC protocols and was approved by their Institutional and Animal Care and Use Committee.

### Infection

Animals were anesthetized with isoflurane and infected intranasally with rat-adapted influenza virus (RAIV) as a 10^−2^ dilution of the stock virus (approximately 2×10^5^ plaque forming units) in a volume of 200 µl on day 0. *Streptococcus pneumoniae* Type 14 was inoculated into brain heart infusion (BHI) broth (day prior to infection) and incubated overnight at 37°C/5% CO_2_. On the day of infection, optical density (575 nm) was determined to confirm growth. Bacteria were subcultured, centrifuged and resuspended in Dulbecco’s phosphate buffered saline (D-PBS). All animals were anesthetized with isoflurane and infected intranasally with *S. pneumoniae* Type 14 (approximately 1×10^7^ colony forming units [CFU] per rat) on experimental Day 28.

### Influenza Antibody Quantification

Blood was collected to measure influenza-specific immunoglobulins IgM and IgG prior to infection with RAIV (Day -8) and post-infection to RAIV (Day 8 for IgM and Day 21 for IgG). Influenza-specific pulmonary IgM and IgG concentrations were measured by enzyme-linked immunosorbent assay (ELISA). Plates were coated with influenza A/Port Chalmers/1/73 (H3N2) virus. Standards, controls, and samples from test animals were added to the pre-coated plates. After washing to remove unbound immunoglobulin, goat anti-rat IgM and rabbit anti-rat IgG HRP conjugated (Bethyl, Montgomery, TX) detection antibodies were added. Unbound conjugated antibodies were removed by washing and the amount of conjugate remaining in the well was measured following incubation with a TMB chromogenic substrate (Zymed, Invitrogen). The resulting absorbance was obtained using a Spectramax 340 microplate reader (Molecular Devices). All samples were run in duplicate and data analysis performed using Softmax Pro v2.2.1 software (Molecular Devices). Relative titers were interpolated from a total rat IgM and IgG standard curve and reported as the mean of duplicate samples. The baseline level of IgM antibody observed in the serum at Day -8 represents the assay background and not influenza-specific antibody.

### Natural Killer Activity

Blood was collected on experimental Day 2 following RAIV infection to assess natural killer cell activity. Target YAC-1 cells were labeled with Chromium-51 (^51^Cr). Effector cells were obtained from whole blood using Ficoll-Paque centrifugation and adjusted to achieve the desired effector-to-target ratios of 25∶1. Effector and target cells were added in triplicate to wells of round-bottom microtiter plates. Spontaneous-release (S) and total ^51^Cr release (T) controls were prepared separately by adding target cells in prepared media (RPMI 1640 or Triton X-100, respectively) to the control wells. The plates were centrifuged to initiate cell contact and subsequently incubated at 37°C/5%CO_2_ for 4 hours. Plates were centrifuged and supernatants harvested and counted with a Cobra II Auto-Gamma counter (Packard Instruments). Percent specific ^51^Cr release (proportional to NK lytic activity) was calculated using the formula [(E−S)/(T−S)]×100; where E is the ^51^Cr release from target cells in the presence of effector cells, S is the spontaneous release of ^51^Cr from target cells alone, and T is the maximum release of ^51^Cr from target cells in the presence of Triton X-100.

### IFNγ Quantification by ELISpot

Blood was collected on experimental Day 8 following RAIV infection to measure IFNγ production as a function of peripheral blood mononuclear cells (PBMCs). PBMC production of IFNγ was determined using a commercial ELISPOT kit (R&D Systems, Inc.; Minneapolis, MN). Blood was collected into K2 EDTA blood collection tubes followed by PBMC isolation using Ficol-Paque centrifugation. Cells were washed and resuspended in complete RPMI 1640 at 1×10^6^/mL. PBMCs (2×10^5^/well) were added to triplicate wells of an ELISpot plate that was pre-coated with anti-rat IFNγ antibodies by the manufacturer. Cells were incubated in the presence or absence of Concanavalin A (ConA, 2.5 mg/mL) for 20 hours. The ELISPOT assay was then performed according to the manufacturer’s instructions and IFNγ-positive spots were counted using an Immunospot® analyzer (CTL-Cellular Technology Limited, Shaker Heights, OH).

### Bacterial and Viral Titration

Animals were euthanized with CO_2_. Long-Evan control animals were euthanized 1 hour post-infection on Day 28 to confirm infection with *S. pneumonia*. LRRK2 KO and age-matched controls were euthanized on Day 29 (24 hours post-infection with *S. pneumoniae*) to evaluate bacterial clearance. Lung homogenates were prepared for determination of *S. pneumoniae* titers. Whole lung homogenates were diluted in PBS and the dilutions plated, each in duplicate, on blood agar plates. Plates were incubated at 37°C/5% CO_2_ for 16–48 hours. Bacterial colonies were counted to determine the number of colony forming units (CFU). The spleen from each animal was dissected in half and preserved for flow cytometric immunophenotyping, as described above. An aliquot of the lung homogenate was cleared by filtration using 0.2 mm syringe filters to remove the bacteria and used to determine influenza virus titers using Madin Darby Canine Kidney (MDCK) cells. Dilutions of filtered lung homogenate were added to monolayers of MDCK cells and covered with an agarose overlay. Following incubation for 36–48 hours to allow plaque development, the cell monolayers were fixed with buffered formalin and stained with crystal violet. Viral plaques were counted visually to determine infectious virus titer.

### Network and Pathway Analysis of LRRK2 Knockout Phenotype

Metabolic and renal data, generated above, was used to assess potential LRRK2 networks and pathways that may help identify potential hypotheses for the observations. Network reconstruction and ontological analysis were performed in MetaCore™ (Thomson Reuters Inc., Carlsbad, CA) with the Systems Toxicology Module. MetaCore™ comprises databases of chemicals, genes, proteins, and their molecular interactions (drug-protein, protein-protein, protein-enzymatic reaction, compound-enzymatic reaction) and biological, toxicological and disease associations for genes, proteins and compounds in the global interactome network. Molecular interaction data was derived from full-text literature curation of experimental data that permits interactome reconstruction based on high confidence interactions using directionality (a→b), mechanism (e.g. binding, phosphorylation, transcriptional regulation) and the nature of the interaction (positive effect or negative effect) [25], [26]. The Systems Toxicology Module further adds a comprehensive ontology of toxic pathologies populated by full-text annotation from the scientific literature with DNA, RNA, protein, and metabolite changes associated with the specific pathological effects of particular compounds, alongside chemical compounds causing the specific toxicity [27]. These data were used to generate interaction networks linking proteins to molecular biomarkers of pathology. Components of ontological categories were also used to query gene or protein lists, experimental data, or networks for overrepresentation with components of particular biological functions, diseases, or pathological states using the hypergeometric distribution [28].

### Statistical Analysis

Two-tailed Student T-tests were used to assess significance between knockout and wildtype control genotypes. Microsoft Excel (2007) was used to perform these analyses.

## Results

### LRRK2 Deficient Rats Display Significant Weight Gain Compared to Wild Type Control Animals

**Figure 1:**
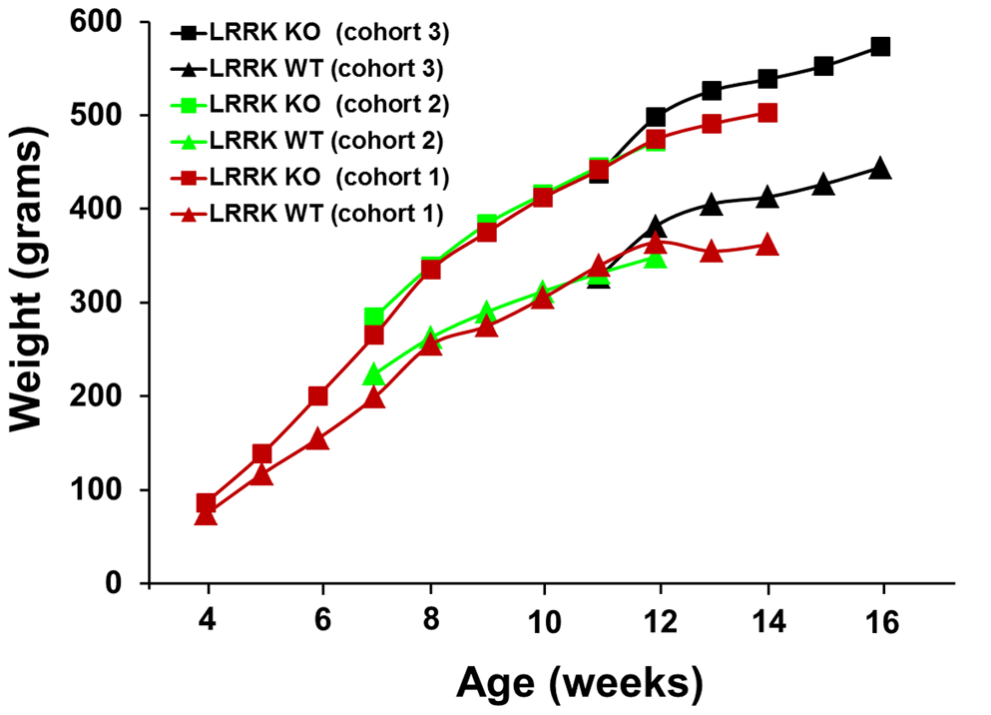
Several cohorts of LRRK2 knockout rats exhibit significant increases in body weight compared to age-matched wild type animals. The average body weight for 5 male LRRK2 knockout and age-matched wild type control animals from three separate cohorts is presented. Significant weight differences were identified amongst cohorts at every time point (P-value <0.05; student’s T-test). Statistical analysis was not conducted between cohorts. Error bars were intentionally removed to enable for a better view of the differences for each of the cohorts.

Weight measurements from several cohorts of rats demonstrated that male LRRK2 KO rats are significantly heavier than their age-matched wild type counterparts (Figure 1 & Figure S1). Significant differences between the genotypes were observed for every week that body weight was measured (analysis performed within each cohort only, P-value <0.05; two-tailed Student’s T-test). Of particular note is that the body weight difference is highly significant even at 4 weeks of age (WT = 74.2±2.4 grams; KO = 86.4±3.0 grams). Group water and food consumption were not significantly different between genotypes when adjusted for group body weight (Table S1). When normalized for body weight, the relative wet tissue weights for the GI tract, kidney and spleen were significantly smaller (P-value = 0.01, 0.02 and 0.01, respectively) in the LRRK2 deficient male rats than the age-matched wild type comparators (Table 1).

**Table 1:** Wet tissue and relative wet tissue weights for LRRK2 wild type and knockout male rats.

	Wet Tissue Weight (grams)			Relative Wet Tissue Weight (% of BW)		
	WT	KO	P-Value	WT	KO	P-Value
Adrenals	0.05±0.02	0.1±0.01	0.27	0.01±0.01	0.01±0.002	0.32
Heart	1.3±0.2	1.6±0.2	0.01	0.4±0.01	0.3±0.03	0.06
GI	20.3±3.5	20.2±2	0.31	5.6±0.3	4.1±0.03	0.01
Kidney	2.9±0.5	3.7±0.3	0.04	0.84±0.1	0.76±0.1	0.02
Liver	14.0±2.5	20.8±1.8	0.004	3.9±0.3	4.3±0.3	0.09
Lung	2.5±0.9	3.0±0.4	0.14	0.7±0.3	0.6±0.1	0.29
Testes	2.9±0.3	3.6±0.5	0.03	0.82±0.07	0.74±0.08	0.07
Thymus	0.5±0.3	0.5±0.2	0.43	0.15±0.09	0.11±0.05	0.16
Spleen	0.8±0.1	0.9±0.05	0.09	0.23±0.03	0.18±0.01	0.01

The average wet tissue weights and relative wet tissue weights are presented (N = 5 male LRRK2 wild type and deficient rats; a two-tailed Student’s T-test was used to assess significance between genotypes; standard deviations of the mean are provided).

GI: Gastrointestinal; Testes: Testicles.

### LRRK2 Deficient Rats Display a Number of Metabolic and Clinical Pathological Perturbations Compared to Wild Type Control Animals

A panel of 21 plasma-based metabolic parameters was assessed in 5 male rats of each genotype (Table 2). A number of significant alterations in the LRRK2 deficient rats were observed, including significant decreases in adiponectin, adrenocorticotropic hormone, and prolactin (P-values = 0.04, 0.01 and 0.01, respectively), whereas there were significant increases in galanin, insulin, insulin-like growth factor, leutenizing hormone, plasminogen activator inhibitor 1, secretin and testosterone (P-values = 0.03, 0.04, 0.05, 0.03, 0.01, 0.02 and 0.004, respectively). Levels of glucagon, glucagon-like peptide 1 and peptide YY were below the levels of quantification for both genotypes. Toxicologically relevant changes in plasma clinical chemistry and hematology assessments (Tables 3 and 4) were not obvious; however there were a number of statistically significant changes between the LRRK2 deficient male rats compared to age-matched wild type control animals. Liver transaminases (ALT and AST) were significantly decreased in LRRK2 deficient rats (p-value = 0.001 and 0.01, respectively), as were triglycerides (p-value = 0.01), total bilirubin (p-value = 0.005), potassium (p-value = 0.03) and chloride (p-value 0.04; Table 3). Cholesterol and albumin were significantly increased (p-value <0.001 and 0.02, respectively; Table 3). Hematologic changes included slight decreases in erythrocytic parameters, absolute lymphocytes and absolute eosinophils in LRRK2 deficient male rats compared to age-matched wild type control animals (Table 4).

**Table 2:** Metabolic analysis of LRRK2 wild type and knockout male rats.

Endpoint Measured	WT	KO	P-Value
Adiponectin (µg/mL)	27.8±9.5	17.6±4.4	0.04
Adrenocorticotropic Hormone(pg/mL)	1013±232	629±286	0.01
Angiotensin-Converting Enzyme(ng/ml)	51.9±8.5	54.2±9.4	0.4
Angiotensin (µg/mL)	266±48.5	306±19.6	0.1
Complement C3α des arg (ng/mL)	602±142	575±115	0.4
Cortisol (ng/mL)	227±17.2	216±21.8	0.2
Galanin (ng/mL)	0.3±0.2	1.0±0.5	0.03
Glucagon (pg/mL)	LOW	LOW	–
Glucagon-like peptide 1 (pg/mL)	LOW	LOW	–
Growth hormone (GH)	50.4±48.3	33.6±17.6	0.2
Insulin (ulU/mL)	29.1±10.5	55.5±29.5	0.04
Insulin-like growth factor (ng/mL)	713±89.7	840±62.1	0.05
Leptin (ng/mL)	2.5±0.9	2.9±0.3	0.2
Lutenizing hormone (ng/mL)	0.5±0.1	0.7±0.2	0.03
Peptide YY (pg/mL)	LOW	LOW	–
Plasminogen activator inhibitor 1 (ng/mL)	0.9±0.2	1.2±0.3	0.01
Progesterone (ng/mL)	50.9±8.8	47.3±9.6	0.2
Prolactin (ng/mL)	42.9±18.9	23.1±13.9	0.01
Resistin (ng/mL)	3.7±0.7	4.6±0.9	0.08
Secretin (ng/mL)	0.8±0.6	2.8±1.1	0.02
Testosterone (ng/mL)	3.0±1.0	4.4±1.6	0.004

The average levels of peripheral blood metabolic parameters are presented (N = 5 male LRRK2 wild type and deficient rats; a two-tailed Student’s T-test was used to assess significance between genotypes; standard deviations of the mean are provided).

**Table 3:** Clinical chemical analysis of LRRK2 wild type and knockout male rats.

Endpoint Measured	WT	KO	P-Value
ALP (U/L)	196±29.1	204±23.5	0.3
ALT (U/L)	75.2±7.6	53.4±4.2	0.001
AST (U/L)	65.8±8.3	56.2±3.9	0.01
BUN (mg/dL)	17.8±1.3	18.8±3.3	0.3
TP (g/dL)	6.4±0.1	6.5±0.2	0.2
CHOL (mg/dL)	94.8±8.3	148±5.9	<0.001
TRIG (mg/dL)	218±50.7	140±61.8	0.01
CRT (mg/dL)	0.3±0.1	0.4±0.1	0.1
GGT (U/L)	14±1	14.4±1.3	0.3
TBIL (mg/dL)	0.6±0.1	0.3±0.1	0.005
PHOSPH (mmol/L)	8.9±0.5	8.7±0.7	0.2
ALB (g/dL)	3.7±0.0	3.8±0.1	0.02
K (mmol/L)	7.4±0.7	6.5±1.2	0.03
CL (mmol/L)	99.9±1	98.1±1.8	0.04
NA (mmol/L)	144±1.1	146±2	0.07
CA (mg/dL)	10.8±0.2	10.9±0.2	0.1

The average plasma clinical chemistry levels are presented (N = 5 male LRRK2 wild type and deficient rats; a two-tailed Student’s T-test was used to assess significance between genotypes; standard deviations of the mean are provided).

ALP: Alkaline phosphatase; ALT: Alanine aminotransferase; AST: Aspartate aminotransferase; BUN: Blood urea nitrogen; TP: Total protein; CHOL: Cholesterol; TRIG: Triglycerides; CRT: Creatinine: GGT: Gamma-glutyamyl transpeptidase; TBIL: Total Bilirubin; PHOSPH: Phosphate; ALB: Albumin; K: Potassium; CL: Chloride; NA: Sodium; CA: Calcium.

**Table 4:** Complete blood count (CBC) analysis of LRRK2 wild type and knockout male rats.

Endpoint Measured	WT	KO	P-Value
WBC (K/µL)	9.5±1.2	7.5±1	0.013
RBC (Millions/µL)	8.6±0.1	7.8±0.3	<0.01
HGB (GM/DL)	16.4±0.8	15.1±0.6	<0.001
HCT (%)	52.6±1.3	48.1±1.7	<0.001
MCV (U^3^)	61±1	62.2±1.8	0.1
MCH (UUG)	18.9±0.8	19.5±0.7	0.1
MCHC (%)	31.1±0.9	31.4±0.4	0.2
NRBC (/100 WBC)	0	0	–
Neutrophil SEG (%)	12.6±4.4	13.8±2.2	0.3
Lymphocyte (%)	84.8±3.6	83.8±2.2	0.3
Monocyte (%)	1.6±1.3	1.4±0.5	0.4
Eosinophil (%)	1	1	–
Basophil (%)	0	0	–
Absolute neutrophils (/µL)	1231±544	1032±167	0.2
Absolute lymphocytes (/µL)	7993±793	6325±906	<0.01
Absolute monocytes (/µL)	140±91	107±48	0.2
Absolute eosinophils (/µL)	94.6±12.2	75.4±9.9	0.01

The average complete blood count levels are presented (N = 5 male LRRK2 wild type and deficient rats; a two-tailed Student’s T-test was used to assess significance between genotypes; standard deviations of the mean are provided).

WBC: White blood cells; RBC: Red blood cells; HGB: Hemoglobin; HCT: Hematocrits; MCV: Mean corpuscular volume; MCH: Mean corpuscular hemoglobin; MCHC: Mean corpuscular hemoglobin concentration; NRBC: Nucleated red blood cells.

### LRRK2 Deficient Rats Display Kidney Morphological and Histopathological Alterations

**Figure 2:**
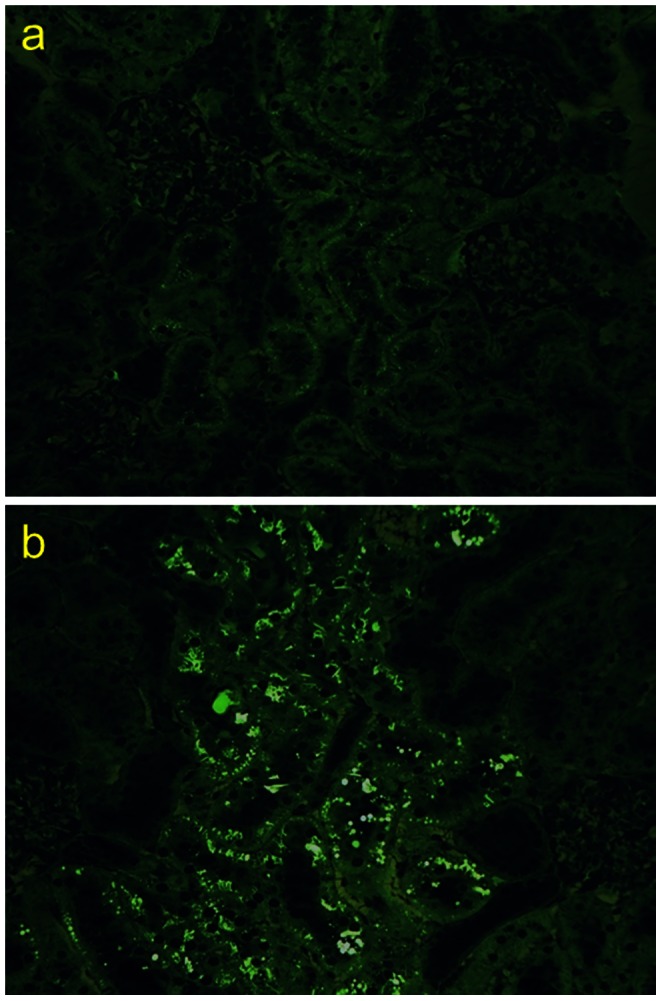
Autofluorescent hyaline droplets are observed in LRRK2 KO, but not wild type rat kidneys. Fluorescent microscopic analysis of kidney proximal tubules reveal no autofluorescence in LRRK2 wild type male kidneys (A) whereas large levels of autofluorescence is readily apparent in LRRK2 deficient kidneys (B).

Kidneys of male LRRK2 KO rats exhibited gross morphological changes, being larger and darker in color than those from wild type animals. Histopathological analysis revealed the presence of eosinophilic hyaline droplets in the renal cortical epithelium and proximal convoluted tubules of all KO rats, but none of the wild type rats. When viewed with fluorescence microscopy, these droplets were autofluorescent (Figure 2). Follow-up analyses were negative for iron (Perl’s stain) and lipid (Oil Red O stain). Urinary and serum kidney biomarker analysis identified several significant alterations associated with the LRRK2 KO genotype (Table 5), with calbindin and GST-α being significantly increased in KO rats (p-value = 0.03 and 0.05, respectively) whereas cystatin-C was significantly decreased (p-value = 0.03). Lipocalin-2 (NGAL) was profoundly decreased (p-value <0.001) in both the urine and serum of the knockout animals with concentrations in the urine below the limit of detection for three out of the five of the knockout animals assessed. No histopathological findings were noted in either the lung or bone marrow of LRRK2 knockout animals.

**Table 5:** Urinary and serum kidney biomarker analysis of LRRK2 wild type and knockout male rats.

	Urine			Serum		
	WT	KO	P-Value	WT	KO	P-Value
Albumin (ng/ml)	3.6±4.9	4.1±6.1	0.9			
Lipocalin-2/NGAL (ng/ml)	0.07	0.0008	<0.001	38.6±4.3	11.5±7.7	<0.001
Osteopontin (ng/ml)	4×10^−5^	2.5×10^−5^	0.2	11.5±1.1	13±5.4	0.3
KIM-1/TIM-1 (ng/ml)	8.7×10^−6^	1.3×10^−5^	0.07	0.1±0.01	0.04±0.01	0.08
β-2 Microglobulin (µg/ml)				65.7±17.3	67.5±20.7	0.4
Calbindin (ng/ml)				0.2±0.1	0.5±0.1	0.03
Clusterin (µg/ml)				144±16.5	172±38	0.08
Cystatin-C (ng/ml)				1104±189	920±162	0.03
GST-α (ng/ml)				680±406	1126±339	0.05
GST-µ (µg/ml)				4.2±2.9	3.9±2.4	0.3
TIMP-1 (ng/ml)				8.3±0.6	7.5±1.2	0.1
VEGF-α (pg/ml)				252±95	224±111	0.2

The average levels of urinary and serum kidney biomarkers are presented (N = 5 male LRRK2 wild type and deficient rats; a two-tailed Student’s T-test was used to assess significance between genotypes; standard deviations of the mean are provided).

NGAL: Neutrophil gelatinase-associated lipocalin; KIM1: Kidney injury molecule 1; TIM1: T-cell immunoglobulin mucin 1; GST-α & -µ: Glutathione S-transferase-α & -µ; TIMP-1: Tissue inhibitor of metalloproteinase 1; VEGF-α: Vascular epilethilial growth factor-α.

### LRRK2 Deficient Rats Exhibit Alterations in the Cellular Composition of their Spleens

**Figure 3:**
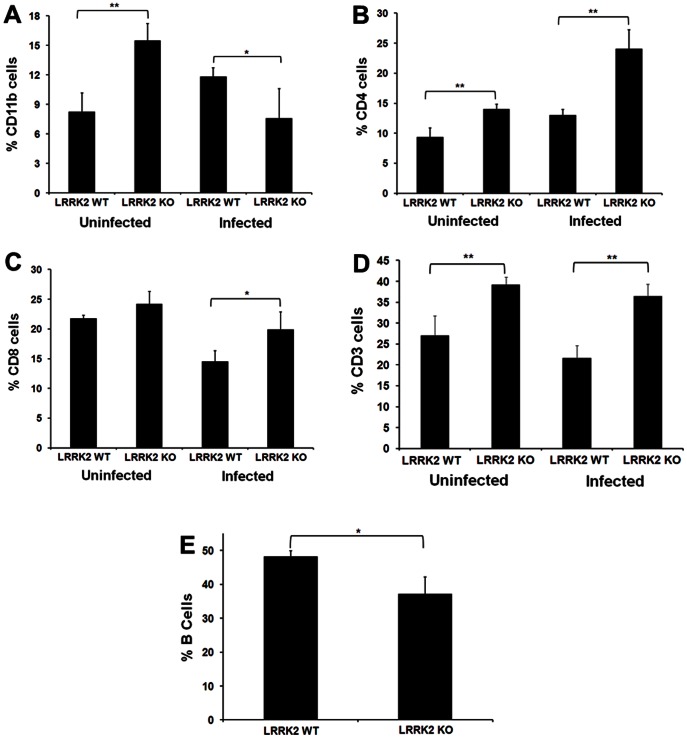
Flow cytometric immunophenotyping of male LRRK2 wild type and deficient spleenocytes. The average percentage of splenocyte cells staining for (A) CD11b, (B) CD4, (C) CD8 and (D) CD3 in uninfected and infected male rats is presented. B-cell data is from uninfected animals only (N = 4 male LRRK2 wild type and deficient rats in uninfected animals and N = 5 in infected animals; a two-tailed Student’s T-test was used to assess significance between genotypes where * represents a P-value <0.05 and ** represents a P-value <0.01; standard deviations of the mean are provided).

Flow cytometric splenocyte immunophenotyping of LRRK2 deficient and wild type male rats, under unstressed conditions as well as following infection with a rat-adapted influenza virus (RAIV) and *S. pneumoniae*, demonstrated that significant differences in the cellular composition exists between the two genotypes (Figure 3). Under normal, uninfected, conditions, there were significantly higher percentage of cells that stained positive for CD11b (Figure 3A), CD4 (Figure 3B) and CD3 (Figure 3D) in LRRK2 KO animals. The relative percent of splenocyte B-cells, however, was significantly lower in LRRK2 KO animals (Figure 3E). Significant alterations in the cellular composition of the spleen were also observed following infection. Specifically, there were significantly lower percentage of cells staining for CD11b (Figure 3A) in LRRK2 KO animals whereas these animals exhibited significantly elevated relative numbers of CD4, CD8 and CD3 positive cells compared to age-matched wild type controls (Figures 3B–D). An analysis exploring the differences between uninfected and infected animals amongst the same genotype was not conducted because a different cohort of animals was utilized for each experiment.

### LRRK2 Deficient and Wild Type Rats Exhibit Comparable Immunological Responses Following Exposure to Rat-adapted Influenza Virus and *Streptococcus pneumoniae*

**Figure 4:**
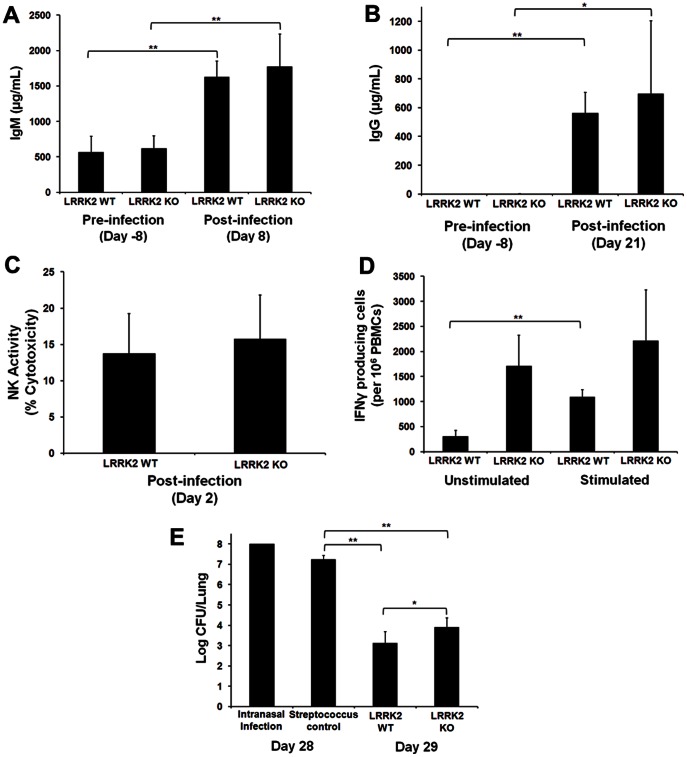
Functional immunological response to rat-specific influenza virus and *Streptococcus pneumoniae*. The average level of serum influenza-specific IgM and IgG were measured in male LRRK2 and deficient rats preceding and following influenza infection (A and B). Average natural killer cell activity, as a function of target cell lysis, was assessed in male LRRK2 and deficient rats on the second day following influenza infection (C). The average number of interferon γ producing cells as a function of peripheral blood mononuclear cells was assessed in male LRRK2 and deficient rats 8 days following influenza infection (D). The average number of lung *Streptococcus pneumonia* bacterial colonies was assessed in male LRRK2 and deficient rats 24 hours following infection (on Day 29), streptococcus control animals were assessed 1 hour following infection on Day 28 (N = 5 male LRRK2 wild type and deficient rats; a two-tailed Student’s T-test was used to assess significance between genotypes where * represents a P-value <0.05 and ** represents a P-value <0.01; standard deviations of the mean are provided).

No influenza-specific IgM or IgG were present in the serum of either genotype prior to infection with RAIV (Figures 4A and B). One week post-infection (Day 8, peak IgM response to influenza), influenza-specific levels were significantly elevated for both genotypes, with no significant differences between genotypes being noted (Figure 4A). Three weeks post-infection (Day 21, peak IgG response to influenza), influenza-specific IgG levels were significantly elevated for both genotypes, although there was no discernible difference between genotypes (Figure 4B). In addition, no significant differences were identified amongst genotypes for natural killer (NK) cell activity two days post-infection when NK activity is known to peak in influenza infected rats (Figure 4C). The average number of interferon-γ producing cells was however noticeably elevated in un-stimulated LRRK2 KO rats compared to the wild type (1710±617 compared to 305±120), though this did not reach statistical significance (Figure 4D). Following stimulation using conA, a significant increase in the average number of interferon-γ producing cells was observed in wild type, but not knockout animals (Figure 4D). Rats were further infected with *Streptococcus pneumoniae* on experimental Day 28 and assessed for the number of lung colony forming units on Day 29. Both the LRRK2 wild type and knockout rats cleared greater than 4 logs of the bacteria that were intranasaly injected the previous day, demonstrating efficient clearance that was consistent with historical data for this model (Figure 4E). Of note is that a small, but significantly elevated number of viable bacteria were present in the lungs of LRRK2 deficient rats relative to the number of viable bacteria present in the lungs of WT rats (Figure 4E). RAIV was not identified in either genotype on Day 29, a finding consistent with historical data for this model.

### Interaction of LRRK2 with the Thioredoxin Pathway is Implied in the Metabolic and Kidney Phenotypes Observed with LRRK2 Deficient Rats

**Figure 5:**
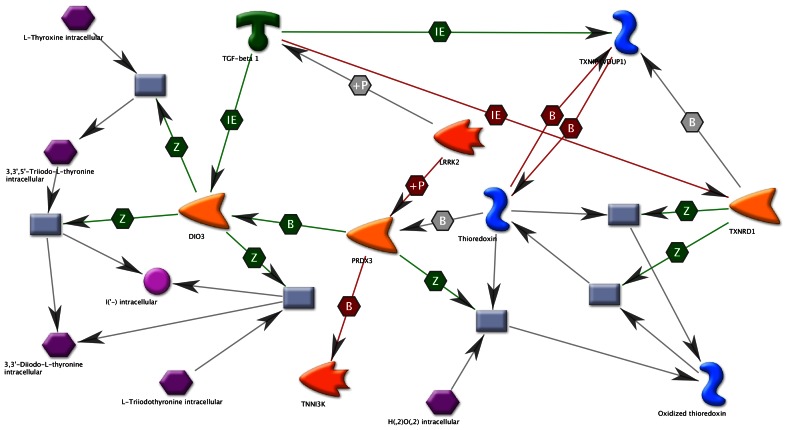
Direct interaction network of LRRK2 with genes associated in the MetaCore disease ontology with nutritional and metabolic disorders. Compounds are represented by hexagons, proteins by solid shapes representing different classes of compound, and enzymatic reactions by gray rectangles. Protein-protein, compound-protein and compound-reaction interactions are shown as unidirectional arrows, and a mechanism of interaction represented by letters in hexagonal boxes over the arrows.

A local neighborhood network around LRRK2 was generated in MetaCore using the *Autoexpand* network algorithm. Enrichment analysis of this network against the Systems Toxicology Module *Toxic Pathologies* ontology indicated a statistically-significant enrichment for biomarkers of kidney pathology, with 8 of the top 10 enriched pathology terms concerning kidney outcomes (Table 6). The kidney pathology biomarker components of the network were thioredoxin, PKC-alpha, angiopoetin 2, TGF-beta, and endothelin receptor type b (EDNRB). Direct links between LRRK2 function and the development of obesity were also revealed by this analysis (Figure 5). Several components of the thioredoxin system are closely linked to LRRK2, including PRDX3, TXNIP, and TXNRD1, which have been identified in recent years as risk factors for obesity and diabetes in human subjects, and as nutrient sensing mechanisms and controlling factors of adipogenesis in mice [29]–[31]. LRRK2 deficiency can also be directly linked, via PRDX3, to the potential dysregulation of iodothyronine deiodinase, type III (DIO3), plausibly leading to hypothyroidism via conversion of T4 and T3 to inactive derivatives.

**Table 6:** Toxic pathology biomarker categories that are over-represented for LRRK2 in the local protein interaction network.

Toxic Pathology	p-value
Kidney-glomerular hypertrophy	1.838e-3
Kidney-arteriolitis	9.153e-3
Kidney-nephron hypertrophy	9.153e-3
Kidney-glomerular injury	9.339e-3
Kidney-hypertrophy	9.779e-3
Kidney-vascular injury	3.114e-2
Kidney-hyaline droplet accumulation	5.113e-2
Lung-fibrosis	9.318e-2
Liver-atrophy	9.939e-2
Kidney-vacuolation	1.112e-1

P-values (from the hypergeometric distribution) for enrichment of the 50-node *Autoexpand* network around LRRK2 with biomarkers of toxic pathologies from the MetaCore Systems Toxicology Module database are presented.

## Discussion

The strong genetic association identified between LRRK2 mutations and Parkinson’s disease has captured the attention of the research community [5], [32], with much interest focusing on whether LRRK2 modulation will be a viable strategy for treating the disease [33]. The use of knockout and transgenic animal models may be useful in generating hypotheses for further exploration, particularly in the absence of pharmacologic tools [34], [35]. In the case of LRRK2, several groups observed that mice of different lineages lacking LRRK2 all exhibited perturbations in kidney homeostasis [18]–[20]. Furthermore, emerging evidence suggests that LRRK2 also has an immunological function [22]–[24], the significance of which remains to be elucidated. The main objectives of the current study were to perform a comparative analysis of LRRK2 deficient rats, characterize kidney morphology, histopathology and homeostasis as well as determine if the putative immunological changes have measurable effects on host resistance to infection with viral and bacterial pathogens.

An early observation regarding the LRRK2 KO rats was that they were significantly larger than their age-matched wild type controls, a trend that was repeated across multiple cohorts (Figure 1). This previously unreported finding for LRRK2 deficient animals does not appear to be due to a difference in eating or drinking habits, as relative consumption was similar between the genotypes. Analysis of metabolic parameters in peripheral blood revealed a number of significant changes between genotypes (Table 2), with increases in insulin and concomitant decreases in adiponectin and growth hormone suggesting that the lack of LRRK2 in these rats may cause metabolic hormone dysregulation. Certain clinical pathology changes, in conjunction with weight gain, could be consistent with hypothyroidism, although TSH, T3 and T4 levels, as well as thyroid histopathology, were not evaluated in this study. Therefore, a specific comment on the potential for thyroid perturbation is not possible. Interestingly, a genome-wide mRNA analysis of LRRK2 deficient mice demonstrated significant differences in prolactin and growth hormone transcript levels in the striatum, cortex, kidneys and muscles [36], data consistent with the metabolic findings identified in the current study. Molecular pathway analysis of LRRK2 reveals direct links between LRRK2 and the thioredoxin system (Figure 5), which interacts with PRDX3, TXNIP and TXNRD1, proteins that are associated with nutrient sensing, adiposity and human obesity [29], [37], [38]. An experimental link between LRRK2 and the thioredoxin system has already been established, as mutations in the LRRK2 kinase domain were identified to significantly increase the phosphorylation of PRDX3, decrease peroxidase activity and increase cell death in cultured neuronal cells [39]. The established link between LRRK2 and PRDX3, a protein associated with adiposity, combined with the data presented herein, suggests that LRRK2 deficiency may affect thioredoxin homeostasis, lead to increased TXNIP turnover and elevated levels of adipogenesis. Further experimental testing will however be required before the hypothetical link between the thioredoxin network, LRRK2 deficiency and the observed rat weight gain can definitively be linked.

Perturbations in kidney homeostasis of LRRK2 deficient mice have consistently been described [18]–[20]. Morphologically, the kidneys of knockout mice are depicted as having a dark red color with changes in their relative weights; histopathologically, the kidneys exhibit autofluorescent microvacuoles in the proximal tubule epithelial cells. These mice were generated separately, on different genetic backgrounds, strong evidence to suggest that the absence of LRRK2 indeed has a role in maintaining kidney homeostasis in the mouse. Herein, LRRK2 knockout rats are for the first time described as having similar morphological and histopathological alterations as those in LRRK2 deficient mice (Table 1 and Figure 1), suggesting that LRRK2’s involvement with kidney homeostasis is not isolated to the mouse and may be conserved across species. One unanswered question related to these findings is whether renal function is impaired following this disruption in homeostasis. In this regard, data in the mouse has been sporadic [18], [40]. In the current study, KIM1 and cystatin C were identified to have irregular patterns and do not consistently portray the phenotype of a damaged kidney (Table 5). Previously published data, along with those presented herein, suggest that kidney function, in spite of the morphological and histopathological findings described, is not drastically altered in LRRK2 knockout animals. Further strengthening this point in rat LRRK2 animals is that lipocalin-2 (NGAL) was identified to be significantly lowered by two separate methods (Table 5), which runs contrary to the expectation that LRRK2 deficient rats are experiencing renal damage. Increases in lipocalin-2 mRNA has been identified to be an early responder of nephrotoxicity and has been validated as a common and sensitive responder to tubular injury [41], [42]. The dramatic decrease in lipocalin-2 levels, identified in both the plasma and urine of LRRK2 knockout rats, is a peculiar finding as lipocalin-2 has been identified to be a critical component in innate immunity to bacterial infection [42]. Thus it is thought-provoking to speculate that the dramatic decreases in lipocalin-2 are independent of renal function but rather are related to an alteration of immune homeostasis.

It has been suggested that these renal changes are the result of an impaired autophagy-lysosomal pathway [18]–[20], data that is consistent with LRRK2’s activation of the MEK/ERK pathway and interaction with basal autophagy [43]. Protein interaction network analysis of LRRK2 reveals several putative networks associated with kidney pathology and function, such as thioredoxin, PKC-apha, angiopoietin 2, TGF-beta and EDNRB (Table 6 and Figure 5). It is interesting to speculate on whether the link between LRRK2 and PRDX3 function, which could decrease the ratio of reduced to oxidized thioredoxin and attenuate the ability to reduce protein thiol bridges, could overwhelm the autophagy machinery and lead to the observed microvacoules in the proximal tubule epithelial cells. LRRK2 is highly expressed in the kidney, as are, thioredoxin, thioredoxin reductases and peroxireodoxins, which show cell-type-specific localization in proximal and distal tubular epithelial papillary collecting ducts. A decreased turnover and subsequent accumulation in vesicles of damaged proteins at the renal tubule epithelium due to impaired processing of damaged proteins and/or impaired autophagy could explain the histopathological findings of hyaline droplets in renal tubules of LRRK2 knockout rats and mice. While teasing apart the molecular mechanisms responsible for the phenotype observed in the LRRK2 knockout animals merits additional investigation, a much more practical concern is determining whether pharmacological inhibition of LRRK2 kinase function will mimic those phenotypes and become rate limiting for their development in the clinic.

A firm link between LRRK2 function and the immune system is emerging, with several lines of evidence suggesting a role in microglial and macrophage inflammatory responses [22]–[24], host response to pathogens [44] as well as inflammatory bowel disease [45], [46]. These findings are of potential concern for a drug that would be used for the chronic treatment of a disease, such as Parkinson’s, as they suggest that a LRRK2 inhibitor may impact the immune system and could increase the likelihood of infection. Flow cytometric immunophenotyping of splenocytes from LRRK2 deficient rats revealed that there are significant alterations in the percent of cells comprising the sub-populations of the spleen when compared to the wild type controls (Figure 3). In an uninfected state, LRRK2 KO rats have significantly higher percentage of CD11b^+^, CD4^+^ and CD3^+^ cells, with a non-significant increase in the number of CD8^+^ cells. Interestingly, the percentage of B-cells is significantly decreased. Alterations in the cell population comprising the spleen could be due either to a decrease number of cells comprising certain sub-populations, thus increasing the percentage observed for the other cell types, or may represent an increase in the cell number which is reflected as an increase in the relative percentage. The data we generated cannot distinguish between these two possibilities and will require additional follow-on experiments to better elucidate the cellular composition of the spleen. In an attempt to understand the functional relevance of these cellular changes, the animals were subjected to a dual-infection host-resistance study where a rat adapted influenza virus (RAIV) and *Streptococcus pneumoniae* were administered intranasally on Days 0 and 28, respectively. Flow cytometric immunophenotyping identified several changes that were in contrast to the non-infected cellular changes mentioned above, including a decrease in the percent of CD11b^+^ cells in the LRRK2 KO animals, whereas CD4^+^, CD8^+^ and CD3^+^ cells increased following infection (Figure 3). While there were several significant changes in the cellular composition of the spleen, the difference in their response to infection between the genotypes appears to be subtle but indicative of an active immune response to infection. Influenza-specific IgG and IgM levels, measured post-infection, were not identified to be different between the two genotypes, as both were significantly increased in comparable fashions following influenza infection (Figure 4A & B). Similarly, there was no difference between genotypes in the NK activity measurements nor in the number of interferon producing cells identified following influenza infection (Figure 4C & D). Interestingly, there was a small but statistically significant increase in the number of *S. pneumoniae* colonies isolated from the lungs of LRRK2 KO rats, though it should be mentioned that both the LRRK2 deficient and wild type rats reduce the bacterial load by greater than 4 logs within 24 hours indicating efficient killing of the bacteria (Figure 4E). Thus, immunological alterations in the LRRK2 knockout rat are described in this study, but any negative impact of these alterations on host resistance to infection appears to be subtle. Assessment of additional infection models assessing other endpoints may be important for risk assessment.

The genetic association identified between LRRK2 and Parkinson’s disease has made it a very attractive drug target for the pharmaceutical industry. Knockout and transgenic animal models can provide insight into the types of potential safety liabilities that may be associated with pharmacological inhibition of any given target. In the case of LRRK2 knockout rats, the genotype was associated with significant weight gain as well as a number of metabolic, clinical pathological, renal and immunological perturbations. Subtle, albeit significant, changes were identified between LRRK2 knockout and wild type animals following infection with RAIV and *Streptococcus pneumoniae*. Additional work is needed to determine if LRRK2 pharmacological inhibition mimics the phenotypes described in the knockout animal model and determine if the risk-benefit profile associated with these therapeutics merits exploration in the clinic.

### Conclusion

The phenotype of the LRRK2 null rat is suggestive of a complex biology that influences metabolism, immune function and kidney homeostasis. These data will be informative in understanding the risk-benefit profile of future LRRK2 inhibitors under development for the treatment of Parkinson’s Disease.

## Supporting Information

Figure S1 **Several cohorts of LRRK2 knockout rats exhibit significant increases in body weight compared to age-matched wild type animals (individual graphs containing standard deviations).**
(TIF)

Table S1 **Relative food and water consumption for LRRK2 wildtype and knockout rats.**
(DOCX)
