# Supplementary figures and images for: Leucine-Rich Repeat Kinase 2 (LRRK2)-Deficient Rats Exhibit Renal Tubule Injury and Perturbations in Metabolic and Immunological Homeostasis
Source: PLoS One. 2013 Jun 14;8(6):e66164. doi: 10.1371/journal.pone.0066164 (PMC3682960; doi:10.1371/journal.pone.0066164)

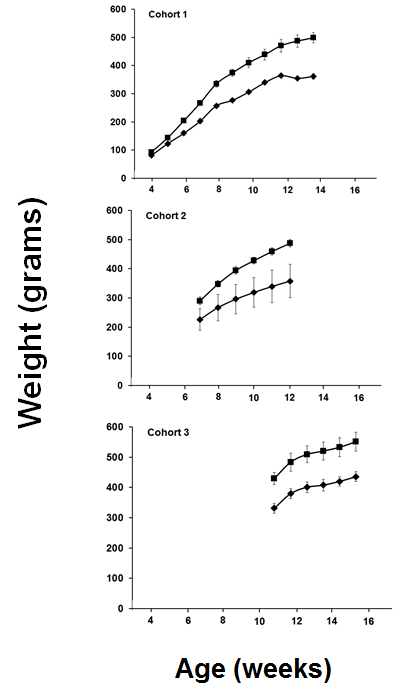

Supplement: Figure S1 — Several cohorts of LRRK2 knockout rats exhibit significant increases in body weight compared to age-matched wild type animals (individual graphs containing standard deviations). (TIF) [file pone.0066164.s001.tif]
