# Supplementary material for: Leucine-Rich Repeat Kinase 2 (LRRK2)-Deficient Rats Exhibit Renal Tubule Injury and Perturbations in Metabolic and Immunological Homeostasis
Source: PLoS One. 2013 Jun 14;8(6):e66164. doi: 10.1371/journal.pone.0066164 (PMC3682960; doi:10.1371/journal.pone.0066164)
Supplement: Table S1 — Relative food and water consumption for LRRK2 wildtype and knockout rats. (DOCX) [file pone.0066164.s002.docx]

Supplemental Table 1: Relative food and water consumption of LRRK2 wildtype and knockout rats (cohort 2)

| **Genotype** | **Animal #** | **Food consumption (g/cage)** | **Water consumption (ml/cage)** | **Rel. food consumption (mg/body weight)** | **Rel. water consumption (ul/body weight)** |
| --- | --- | --- | --- | --- | --- |
| Wildtype | 1 | 13.8 | 28.7 | 45.3 | 94.3 |
| Wildtype | 2 | 18.5 | 32.6 | 60.3 | 106.2 |
| Wildtype | 3 | 14.3 | 31.2 | 32.9 | 71.7 |
| Wildtype | 4 | 13.9 | 30.7 | 39.3 | 86.6 |
| Wildtype | 5 | 18.1 | 39.7 | 46.1 | 101.1 |
| Knockout | 6 | 20.2 | 41.5 | 40.0 | 82.3 |
| Knockout | 7 | 22.7 | 43.4 | 48.1 | 92.0 |
| Knockout | 8 | 18.1 | 44.8 | 38.1 | 94.5 |
| Knockout | 9 | 26.5 | 38.9 | 52.6 | 77.4 |
| Knockout | 10 | 19.0 | 38.1 | 39.9 | 80.0 |
| **WT Average** | | **15.7** | **32.6** | **44.8** | **92.0** |
| **WT Standard Deviation** | | **2.4** | **4.2** | **10.2** | **13.5** |
| **KO Average** | | **21.3** | **41.4** | **43.7** | **85.2** |
| **KO Standard Deviation** | | **3.4** | **2.9** | **6.3** | **7.6** |
